# Epidemiology and molecular characterization of influenza viruses in Burkina Faso, sub‐Saharan Africa

**DOI:** 10.1111/irv.12539

**Published:** 2018-04-24

**Authors:** Armel M. Sanou, Sampoko Carine M. Wandaogo, Armel Poda, Laure Tamini, Anselme E. Kyere, Tani Sagna, Macaire S. Ouedraogo, Maude Pauly, Judith M. Hübschen, Claude P. Muller, Zekiba Tarnagda, Chantal J. Snoeck

**Affiliations:** ^1^ National Influenza Reference Laboratory Institut de Recherche en Sciences de la Santé Bobo‐Dioulasso Burkina Faso; ^2^ Hôpital du jour, Service des maladies infectieuses CHU Souro Sanou Bobo‐Dioulasso Burkina Faso; ^3^ Université Polytechnique de Bobo‐Dioulasso (UPB) Bobo‐Dioulasso Burkina Faso; ^4^ Service de Pédiatrie CHU Pédiatrique Charles De Gaulles Ouagadougou Burkina Faso; ^5^ Unité de Formation et de Recherche en Sciences de la Santé (UFR/SDS) Université de Ouagadougou Ouagadougou Burkina Faso; ^6^ Infectious Diseases Research Unit Department of Infection and Immunity Luxembourg Institute of Health Esch‐sur‐Alzette Luxembourg

**Keywords:** Burkina Faso, children, epidemiology, influenza, surveillance, vaccination

## Abstract

**Background:**

The importance of influenza viruses in respiratory infections in sub‐Saharan Africa has been historically overlooked, including in Burkina Faso.

**Objectives:**

This study therefore aimed at evaluating the prevalence and seasonal occurrence of influenza viruses in children under 5 years old, at risk of influenza‐related complications, presenting with influenza‐like illness (ILI) or severe acute respiratory infection (SARI). The study also aimed at identifying the periods with increased influenza transmission for vaccination recommendations in Burkina Faso.

**Methods:**

From January 2014 to December 2015, ILI and SARI (2015 only) patients were recruited in six healthcare centers in Burkina Faso. Influenza A and B molecular detection and subtyping were performed. Clade clustering of a subset of A(H1N1)pdm09 and A(H3N2) strains was deduced by performing phylogenetic analyses on hemagglutinin gene sequences. Weekly surveillance data from FluNet (2011‐2013; 2016) and this study (2014‐2015) were used to identify periods of increased influenza activity.

**Results:**

Influenza A and B viruses were detected in 15.1% (112 of 743) of ILI and 6.6% (12 of 181) of SARI patients. Overall, influenza A viruses were largely predominant (81 of 124, 65.3%), with 69.1% of A(H3N2) and 30.9% of A(H1N1)pdm09 strains. Four waves of increased transmission were identified in 2014‐2015, each dominated by different influenza subtypes and clades. Between 2011 and 2016, periods of increased influenza activity varied in their frequency, duration, and timing.

**Conclusion:**

Influenza A and B viruses were detected in a substantial number of ILI and SARI cases in Burkina Faso. Vaccination in September‐October would likely protect the highest number of patients.

## 1 BACKGROUND

Despite the burden of influenza infections worldwide,^1^, ^2^ morbidity and mortality associated with influenza have been largely overlooked in sub‐Saharan Africa,^3^ likely due to previous lack of access to diagnostic tools. Misinterpretation of non‐pathognomonic influenza symptoms with those induced by various viral and bacterial respiratory infections or by malaria or Lassa fever also complicates the diagnosis.^4^, ^5^, ^6^, ^7^ The implementation of the WHO Global Action Plan for Influenza Vaccines, strongly promoting seasonal influenza vaccination,^8^ remains challenging in (sub‐)tropical low‐income countries. In contrast to temperate regions usually having a single influenza season, various patterns of circulation (one season, two seasons, or even year‐round) can be observed in tropical regions.^9^, ^10^, ^11^, ^12^ However, proper understanding of influenza seasonality for each country, necessary for successful vaccination, is still lacking.

In Burkina Faso, an influenza sentinel surveillance for outpatients with influenza‐like illness (ILI) was established at the National Influenza Reference Laboratory in 2010, in the aftermath of the A(H1N1) pandemic. In a preliminary report covering 2010‐2012, 6.6% of ILI cases in the general population were influenza positive.^13^ The few studies investigating the seasonal circulation of influenza in Burkina Faso were not conclusive on seasonal patterns,^10^, ^13^, ^14^ hindering proper recommendations for a national policy for the management and prevention of influenza‐related illnesses, and no seasonal vaccination is currently performed in Burkina Faso. We therefore initiated a two‐year influenza surveillance in several healthcare facilities in Burkina Faso to assess the prevalence and seasonal occurrence of influenza viruses in children with ILI and severe acute respiratory infection (SARI). Prevalence data gathered in this study were analyzed together with FluNet surveillance information to identify the periods with increased influenza transmission for vaccination recommendations.

## 2 MATERIALS AND METHODS

### 2.1 Ethics statement

This study was approved by the Institutional Ethical Committee of Centre Muraz (Ethic Clearance number 13‐2013/CE‐CM). Samples were collected after written informed consent from parents or legal guardians. Laboratory results were shared with the participating healthcare centers.

### 2.2 Inclusion criteria

For the purpose of this study, children up to 60 months of age presenting with ILI or SARI symptoms were enrolled. ILI cases were defined as outpatients presenting with a history of fever or measured fever (≥38°C) and cough or sore throat, with the onset of symptoms within the prior 10 days. SARI cases were defined as inpatients with a history of fever or measured fever (≥38°C), and cough or difficult breathing, with the onset within the last 10 days.

### 2.3 Data and sample collection

Starting in January 2014, we implemented an active surveillance of patients presenting with ILI in four healthcare centers (Colsama, Accart‐ville, Do and Leila), all situated in Bobo‐Dioulasso, the second most populated city in Burkina Faso after Ouagadougou. In January 2015, the surveillance activities were extended to two other hospitals. Outpatients with ILI, as well as hospitalized children presenting with SARI, were recruited at the hospital of Bogodogo in Ouagadougou. Patients with SARI were also recruited at the Centre Hospitalier Universitaire Souro Sanou (CHU‐SS) in Bobo‐Dioulasso (Table 1). Both hospitals are the referral hospitals for SARI cases in their respective regions (ie, Centre and Hauts‐Bassins regions). All healthcare centers were selected based on the following criteria: geographic accessibility, high number of patients seeking medical care, presence of qualified physicians and nurses willing to participate to the study, and the ability to directly store samples at low temperatures.

**Table 1:** Demographics of patients with ILI or SARI, 2014‐2015

Variable	No. of ILI patients	No. of SARI patients	Total
Gender			
Male	406	112	518
Female	337	69	406
Age groups (in mo)			
0‐6	171	65	236
7‐24	400	88	488
25‐60	172	28	200
Healthcare centers			
Accart‐ville^a^	221	‐	221
Colsama^a^	267	‐	267
Do^a^	65	‐	65
Leila^a^	78	‐	78
University hospital^a^	‐	151	151
Bogodogo^b^	112	30	142

a Healthcare center located in Bobo‐Dioulasso.

b Healthcare center located in Ouagadougou.

The physicians and nurses were trained before the beginning of the study on inclusion criteria, sample collection, and storage. A nasopharyngeal swab was collected from each ILI patient, and a nasopharyngeal aspirate was collected from each SARI patient. All samples were resuspended in viral transport medium (Copan, Italy). Demographic, socio‐economic, and clinical data were collected from all participants using a questionnaire administered by trained personnel.

### 2.4 Influenza virus detection methods, characterization, and phylogeny

Viral nucleic acids were extracted using QIAamp Viral RNA Mini kits (Qiagen, Venlo, the Netherlands). Influenza viruses were detected and typed by real‐time RT‐PCR using the Human Influenza Virus Real‐Time RT‐PCR Diagnostic Panel, Influenza A subtyping, and Influenza B lineage differentiation kits (CDC Influenza Division, Atlanta, USA).

Only influenza A‐positive samples with Ct values ≤30 were selected for full hemagglutinin (HA) gene sequencing. Viral cDNA was synthetized using the universal primer Uni12.^15^ The full‐length HA gene of A(H1N1)pdm09 and A(H3N2) viruses was amplified with (semi‐nested) PCRs using specific primers (primers and PCR conditions available upon request). PCR product purification and sequencing were performed as described before.^16^

Sequences were analyzed and assembled using SeqScape v2.5 software (Applied Biosystems). H1 and H3 sequences (submitted to GISAID under segment IDs EPI1020018‐EPI1020021, EPI1020030, EPI1020039, EPI1020048‐EPI1020063, EPI1020088‐EPI1020108) were aligned with reference sequences as defined by the WHO Collaborative Center for Reference and Research on Influenza, London, as well as representative strains of the clades of interest. All sequences were retrieved from the GISAID database (http://www.gisaid.org). The best fit model was selected for each tree, and phylogenetic analyses were performed using MEGA6 with 500 bootstrap replicates.^17^

### 2.5 Data analysis

Influenza activity was measured as the proportion of positive cases per month. Periods of increased activity were defined as periods of at least 2 months with influenza activity exceeding the annual median proportion of positive cases, separated by at least 1 month with influenza activity below the annual median proportion (CDC approach^10^). Influenza surveillance data from Burkina Faso were retrieved from FluNet database (http://www.who.int/influenza/gisrs_laboratory/flunet/en/) as weekly numbers and aggregated per months (years 2011‐2013, 2016). Because some of the results from 2014 generated by this study were submitted to FluNet while almost no data were available on FluNet for 2015, trends for 2014 and 2015 were assessed based on data generated in this study. For 2014‐2015, the seasonal peaks of influenza transmission were defined as the week with the highest number of influenza‐positive cases during period of increased activity.

Statistical analyses (Chi‐square tests, Mann‐Whitney Rank Sum Tests, and z‐test for low proportions) were performed using SigmaPlot version 12.0 (San Jose, CA, USA). *P*‐values lower than .05 were considered statistically significant.

## 3 RESULTS

### 3.1 Demographic and clinical characteristics of enrolled patients

Between January 2014 and December 2015, a total of 924 children up to 5 years old with ILI (743 of 924; 80.4%) or SARI (181 of 924; 19.6%) were enrolled in six healthcare centers (Table 1). Of all children enrolled, 518 were males (56.1%) and half of all patients were ≤1 year old (50.1%). The mean age of patients enrolled was 17.6 months (ILI: 18.5 months, SARI: 14.0 months, *P* < .001, Mann‐Whitney Rank Sum Test).

### 3.2 Prevalence of influenza viruses and associated factors

Influenza A and B viruses were detected in 13.4% (124 of 924) of the patients. Influenza A viruses were largely predominant (81 of 124, 65.3%; Table 2), with A(H3N2) viruses more than twice as frequent (56 of 81, 69.1%) as A(H1N1)pdm09 viruses (25 of 81, 30.9%). Among influenza B viruses, the B/Victoria (15 of 43, 34.9%) and the B/Yamagata (14 of 43, 32.6%) lineages were found with basically equal frequencies (Table 2). One‐third of influenza B viruses (14 of 43) could not be characterized up to the lineage level. No cases of co‐infection with influenza A and B viruses were detected.

**Table 2:** Type and subtype of influenza viruses detected in ILI (2014‐2015) and SARI (2015) patients

Influenza (sub)type	ILI	ILI	SARI	Total (%)
	2014	2015	2015	
Influenza A	56	18	7	81 (65.3)
A(H1N1)pdm09	13	10	2	25 (30.9)
A(H3N2)	43	8	5	56 (69.1)
Influenza B	27	11	5	43 (34.7)
B/Victoria	15	0	0	15 (34.9)
B/Yamagata	2	8	4	14 (32.6)
B/untyped	10	3	1	14 (32.6)
Total	83	29	12	124

**Figure 1:**
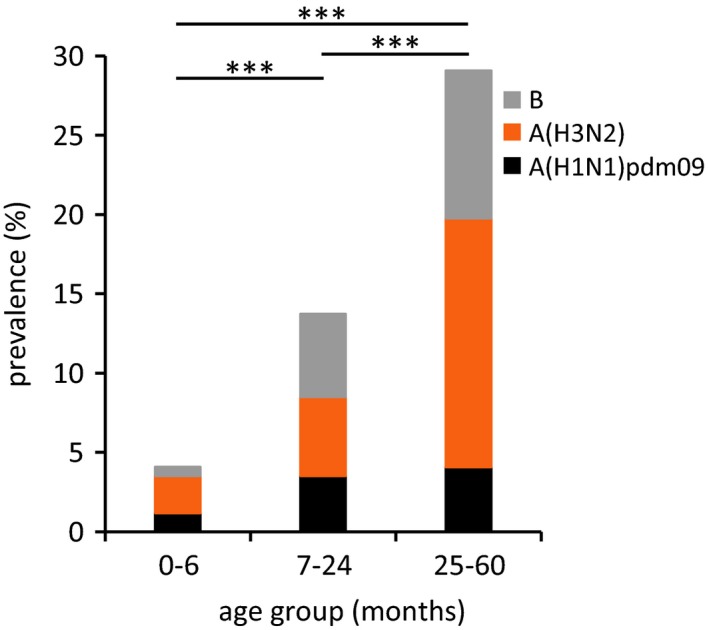
Distribution of influenza‐positive ILI patients according to age groups

Influenza A and B viruses were detected in 112 of 743 ILI (15.1%) and 12 of 181 SARI (6.6%) patients. The detection rate of influenza viruses among ILI patients significantly increased with age (Figure 1). Very young children (0‐6 months; 7 of 171, 4.1%) were significantly less frequently infected by influenza viruses compared to 7‐24 (55 of 400, 13.8%, *P* = .001) and 25‐60 (50 of 172, 29.1%, *P* < .001) months old children. In SARI patients, no association between age and virus detection was possible due to the low number of influenza‐positive cases.

The vast majority of ILI patients (700 of 743, 94.2%) received antibiotics, mainly β‐lactams (492 of 700, 70.3%) or sulfonamides (181 of 700, 25.9%). Most SARI patients (155 of 181, 85.6%) were also treated with antibiotics, mainly with β‐lactams (151 of 155, 97.4%) in combination (40 of 151, 26.5%) or not with other antibiotics. All influenza‐positive SARI patients received antibiotherapy.

### 3.3 Influenza virus circulation in 2014‐2015

**Figure 2:**
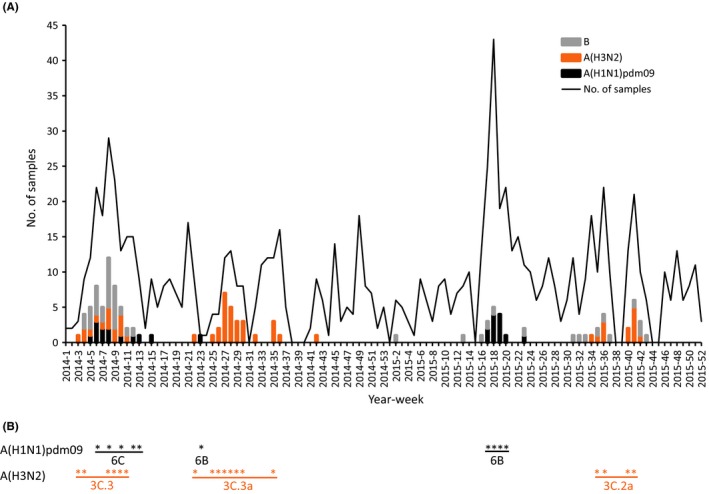
Weekly distribution of the number of samples collected and influenza virus detection by influenza type and subtype from ILI and SARI patients from January 2014 to December 2015 in Burkina Faso (panel A). Temporal repartition of influenza A strain sequenced and used in phylogenetic analyses (panel B). A star corresponds to ≥1 strain sequenced for the corresponding week of panel A

While the number of samples collected per week varied throughout the study period, several waves of influenza virus circulation became apparent (Figure 2). A first wave of transmission was observed between end of January and beginning of April 2014 (weeks 3‐15; peak in week 8) with co‐circulation of A(H1N1)pdm09 clade 6C, A(H3N2) clade 3C.3, and B viruses (Figures 2, S1 and S2). A second wave almost exclusively due to A(H3N2) clade 3C.3a occurred between end of May and mid‐September 2014 (weeks 22 to week 36; peak in week 27). One A(H1N1)pdm09 strain of clade 6B was detected in June. In 2015, a first wave predominantly due to A(H1N1)pdm09 clade 6B was observed between mid‐April and mid‐June (weeks 16‐23; peak in week 18). A(H3N2) clade 3C.2a and B types co‐circulated between August and mid‐October (weeks 31‐43; peak in week 41; Figures 2, S1 and S2), and a single A(H1N1)pdm09 clade 6B.2 was reported in August (Figure S1). Influenza B/Victoria strains (n = 15) were exclusively detected in the first 2014 wave together with 2 B/Yamagata strains. In 2015, B/Yamagata strains (n = 12) were detected sporadically throughout the year (Table 2). The (sub‐)types detected in SARI patients matched those found in ILI patients during the same period. No temporal difference between Bobo‐Dioulasso and Ouagadougou was observed (2015).

### 3.4 Periods of increased influenza activity between 2011 and 2016

Between 2011 and 2016, periods of increased influenza transmission varied between years in number, in time, and in duration. According to the criteria of proportion of influenza‐positive cases above annual median proportion for a minimum of two consecutive months, increased influenza activity was identified in September‐October 2011, February‐March 2012, September 2012‐March 2013, October 2013‐March 2014, June‐August 2014, April‐June 2015, August‐October 2015, and June‐October 2016 (Table 3). On a week‐scale, two clear peaks can be identified for two of three prolonged seasons (>3 months) with different dominant viruses (November 2012 with A(H3N2) and B viruses, and February 2013 with A(H1N1)pdm09; November 2013 with A(H3N2) and February 2014 with all three influenza subtypes; data from FluNet and this study). Over the entire surveillance period (2011‐2016), influenza activity was highest in January‐March and September‐October (Table 3).

**Table 3:** Periods of increased influenza activity in Burkina Faso, 2011‐2016

Month	No. of influenza positives/No. of samples collected per month (proportion of positive cases per month*100)						
	2011^a^	2012^a^	2013^a^	2014	2015	2016^a^	2011‐2016
January	‐	1/69 (1.4)	**14/46 (30.4)**	**10/28 (35.7)**	1/20 (5.0)	0/27 (0.0)	**26/190 (13.7)**
February	‐	**2/43 (4.7)**	**31/57 (54.4)**	**33/92 (35.9)**	0/26 (0.0)	0/58 (0.0)	**66/276 (23.9)**
March	‐	**2/31 (6.5)**	**9/40 (22.5)**	**10/52 (19.2)**	1/28 (3.6)	1/37 (2.7)	**23/188 (12.2)**
April	‐	2/47 (4.3)	0/29 (0.0)	1/24 (4.2)	**9/91 (9.9)**	1/44 (2.3)	13/235 (5.5)
May	‐	0/29 (0.0)	3/61 (4.9)	1/47 (2.1)	**5/69 (7.2)**	0/77 (0.0)	9/283 (3.2)
June	‐	1/32 (3.1)	0/53 (0.0)	**4/10 (40.0)**	**2/35 (5.7)**	**3/55 (5.5)**	10/185 (5.4)
July	0/31 (0.0)	0/37 (0.0)	3/51 (5.9)	**18/41 (43.9)**	1/41 (2.4)	**6/31 (19.4)**	28/232 (12.1)
August	4/49 (8.2)	1/44 (2.3)	3/77 (3.9)	**4/40 (10.0)**	**5/41 (12.2)**	**10/48 (20.8)**	27/299 (9.0)
September	**11/75 (14.7)** b	**3/24 (12.5)**	3/55 (5.5)	1/21 (4.8)	**5/32 (15.6)**	**16/66 (24.2)**	**39/273 (14.3)**
October	**14/72 (19.4)**	**11/24 (45.8)**	**13/65 (20.0)**	1/18 (5.6)	**12/50 (24.0)**	**15/55 (27.3)**	**66/284 (23.2)**
November	1/46 (2.2)	**13/29 (44.8)**	**12/59 (20.3)**	0/26 (0.0)	0/29 (0.0)	0/31 (0.0)	26/220 (11.8)
December	‐	**8/29 (27.6)**	**12/56 (21.4)**	0/35 (0.0)	0/28 (0.0)	0/46 (0.0)	20/194 (10.3)
Total	30/273 (11.0)	44/438 (10.0)	103/649 (15.9)	83/434 (19.1)	41/490 (8.4)	52/575 (9.0)	353/2859 (12.3)
Annual median proportion	8.2	4.5	12.9	7.8	5.4	2.5	11.9

a Data from FluNet.

b Periods of increased influenza activity (activity > annual median proportion for ≥2 mo) are shown in boldface.

## 4 DISCUSSION

Young children are a major risk group for complications due to influenza infection.^18^, ^19^, ^20^ They often represent the majority of patients enrolled in ILI surveillance, also in Africa.^13^, ^21^, ^22^, ^23^, ^24^ In Africa, influenza accounts for 8%‐20% of ILI cases in children below 5 years old^21^, ^22^, ^23^, ^24^, ^25^ and around 7% of SARI cases.^26^ Similarly, we found that 15.1% of ILI and 6.6% of SARI patients enrolled in our study tested positive for influenza A or B viruses over the 2 years period**.** For ILI cases, the prevalence of influenza increased with age, similarly to other studies.^22^, ^23^, ^24^, ^25^ Particularly in very young children, the broad definition of “influenza‐like illness” leads to the inclusion of patients with respiratory infections caused by other pathogens, especially respiratory syncytial virus and rhinoviruses.^27^, ^28^, ^29^ Increasing socialization with the children's age also contributes to age‐dependent differences in risk of infection^30^, ^31^, and lower influenza incidence in children <6 months old may indicate protection by maternal antibodies.^32^, ^33^ Nevertheless pregnant women are the primary target group for influenza vaccination, to avoid complications during pregnancy. It also contributes to protecting their infants during their first 6 months of life when they are at increased risk of severe complications^18^ and yet ineligible for vaccination.^3^, ^20^

Influenza vaccination is not yet implemented in Burkina Faso and will require a proper understanding of the seasonal circulation of influenza viruses that has not reached a consensus yet. Previous surveillance studies during the early implementation of the influenza network in the country reported one peak of increased transmission but varying in timing (December 2010‐February 2011 and August‐October 2011).^13^, ^14^ A meta‐analysis using three different statistical approaches on three non‐comparable FluNet datasets (2011‐2013, 2010‐2014, and 2013‐2014) was not conclusive on the number or on the synchronicity of periods with increased influenza transmission.^10^ By extending the dataset to a total of 6 years (2011‐2016), our results reveal that periods of increased influenza activity vary in timing, in number of occurrences per year, and in duration. Some periods are short (2‐3 months) while others can last for 5‐7 consecutive months. Prolonged influenza seasons may be due to overlapping waves of circulation of different viruses. Also successive waves of influenza circulation in 2014‐2015 were due to viruses from different subtypes and clades, as revealed by phylogenetic analyses of 14 A(H1N1)pdm09 and 29 A(H3N2) strains, suggesting new seeding events at least twice a year and sometimes within short time intervals (Figure 2). Despite a possible lack of sensitivity due to non‐homogenous surveillance efforts, influenza viruses do not seem to circulate year‐round at background levels in Burkina Faso, similarly to Niger (analysis of FluNet data for Niger ‐ not shown ‐ and data from Mainassara et al.^22^) and unlike Côte d'Ivoire, Ghana or Togo,^10^, ^23^, ^25^, ^34^ with a different climate. In the future, increased efforts toward sampling and individual strain characterization will help to better understand the temporal occurrence of influenza activity, virus spread between countries and potential persistence within West Africa.^35^

Although variations across years of surveillance occur, influenza epidemics seem to happen mostly in September‐October, corresponding roughly to the end of the rainy season and beginning of the Harmattan season (colder, dry, windy season), and January‐March, essentially corresponding to the end of the Harmattan season (aggregated data over years, Table 3). Vaccination with the Northern hemisphere vaccine formulation as early as possible in September‐October would cover this prolonged period, as suggested before.^10^ Vaccination in April would have little benefit due to overall low influenza activity in April‐June and waning protection with time.^36^ If strains emerge during unusual periods and/or exhibit a major antigenic drift affecting vaccine‐induced immunity, additional vaccination schedules may be required,^37^ but would be difficult to implement. Until yearly variations are better understood, vaccination of pregnant women could be recommended at any time of the year with the most recent vaccine formulation available.^12^

The vast majority of ILI patients enrolled in this study were treated with antibiotics, despite that viruses are usually the main cause of uncomplicated ILI.^29^ This high proportion, which is not uncommon and not solely an issue of low‐income countries,^38^ highlights the need for revision of clinical best practice to identify cases of bacterial (co)infections.^39^, ^40^ Education and awareness of the healthcare personnel as well as the patients’ parents toward the risks linked to antibiotic resistance will also be necessary to efficiently control over‐prescriptions.^41^, ^42^

## 5 CONCLUSION

Our findings showed that influenza viruses are implicated in a substantial number of ILI and SARI syndromes in children in Burkina Faso, and current data are supporting vaccination in September‐October to decrease influenza burden. The influenza surveillance network in Burkina Faso should be sustained to further refine vaccination recommendations and to evaluate the cost‐benefit of seasonal vaccination of high‐risk patients.

## COMPETING INTERESTS

The authors declare that they have no competing interests.
